# Impact of therapeutic and high doses of florfenicol on kidney and liver functional indicators in goat

**DOI:** 10.14202/vetworld.2016.1135-1140

**Published:** 2016-10-24

**Authors:** Jan Muhammad Shah, Toufique Ahmed Qureshi, Tahmina Shah, Qurban Ali Shah, Muhammad Asif Arain, Zohaib Ahmed Bhutto, Muhammad Saeed, Farman Ali Siyal

**Affiliations:** 1 Department of Pharmacology, Shaheed Benazir Bhutto University of Veterinary and Animal Sciences, Sakrand, Pakistan; 2 Department of Dairy Technology, Baqai College of Veterinary Sciences, Baqai Medical University, Karachi, Pakistan; 3 Department of Animal Husbandry, Faculty of Veterinary and Animal Sciences, Lasbela University of Agriculture, Water and Marine Sciences, Uthal 3800, Pakistan; 4 College of Animal Sciences and Technology, Northwest A & F University, Yangling 712100, China; 5 Department of Animal Nutrition, Faculty of Animal Husbandry and Veterinary Sciences, Sind Agriculture University Tandojam, Pakistan

**Keywords:** blood chemistry, florfenicol, goat, liver and kidney function tests, therapeutic and high doses

## Abstract

**Aim:**

The aim of this study was to evaluate the impact of therapeutic and high doses of florfenicol on kidney and liver functional indicators in goat species.

**Materials and Methods:**

Six mature, healthy goats (combine breed and sex) with average weight 25 kg were selected for this study. The therapeutic (20 mg/kg b.w.) and high doses (40 and 60 mg) of florfenicol were administered for 3 days with 24 h interval. Blood samples were collected at 0, 24, 48, 72, 96, and 120 h following the each administered dose.

**Results:**

The results showed that the therapeutic dose of florfenicol produced nonsignificant effect on serum urea, creatinine, total protein (TP), alkaline phosphatase (ALP), gamma-glutamyl transferase (GGT) and bilirubin on all timings, and increased (p<0.05) the serum glutamic oxaloacetic transaminase (SGOT) and serum glutamate-pyruvate transaminase (SGPT) levels for 48 h. Whereas the high doses of florfenicol (40 and 60 mg) significantly altered the kidney and liver functional indicators in the blood. In contrast with control, the serum urea level was (p<0.01) increased at all timing points. Creatinine values were altered (p<0.01, <0.05) in increasing manner from 24 to 96 h. The high dose of 40 mg decreased the TP (p<0.05) for 72 h and 60 mg persisted same effect (p<0.01) up to 120 h. The indices of ALP, GGT, SGOT, and SGPT were raised (p<0.01, <0.05) at all timings. The bilirubin indexes also (p<0.05) elevated from 48 to 72.

**Conclusion:**

It was concluded that the high doses of florfenicol produced reversible dose-dependent effects on functional indicators of kidney and liver such as urea, creatinine, TP, ALP, SGOT, SGPT, GGT, and bilirubin.

## Introduction

Chloramphenicol is broad spectrum antibiotic compound, was employed to curing the numerous infectious diseases caused by bacteria. Florfenicol belonging to amphenicols are broad-spectrum antibiotics commonly used in veterinary as well as in aquaculture to treat the infections caused by bacteria [1,2]. These types of antibiotics bind permanently to the 50S ribosomal subunit to inhibit the protein synthesis [1]. Nowadays, these antibiotics are restricted simply for specific infectious diseases in veterinary clinics because of their noxious effects on various organs and body systems such as excretory and hepatic. They were also eradicated due to prolong persistence of their metabolites and residues in the body resulting long-term toxicity [3]. It was a vital requirement to find out novel drugs which would have the similar efficacy in treatment with less adverse effects. Florfenicol is the drug of choice in veterinary field for curing the domestic animals due to its harmless and less toxic properties [4]. Florfenicol is fluorinated derivative of chloramphenicol, acquire the similar features of the parent compound, however, is less blamed to produce harsh and unfavorable effects [5,6]. The florfenicol can be used as an alternative to chloramphenicol. In the body, less amount of florfenicol metabolized due to this reason various high doses of florfenicol were under trial at clinical and laboratory levels [3]. The therapeutic dose of florfenicol produce reversible short-term toxic effects on the kidney and liver functional indices in the piglets. With time after cessation of florfenicol administration these effects gradually return to normal [7]. The recent studies proved that florfenicol can be used as a possible synergistic antimicrobial modulator along with many kinds of antibiotic and is effectively kill both Gram-positive and Gram-negative depending on its combination with antibiotic [8]. Previously documented research proved that high dosages of florfenicol produced dose-dependent toxicities such as alteration in kidney and liver functional indicators in various species, in mice [9], horse [10], pig [3,11], chicken [12], and in fish [13], To avoid these toxic effects throughout therapy with high doses, laboratorial periodic estimation of kidney and liver indices should be carried out to get clinically useful stable serum and tissue concentration of drug for prolonged period.

The little is acknowledged about different high doses of florfenicol along with their influence on nephro and hepato toxicity in small ruminants particularly in goat. Although many research reports about the cytotoxicity with high doses of florfenicol, limited studies were found on the liver and kidney functional indicators in farm animals.

The florfenicol was used to treat numerous infectious diseases caused by bacteria. However, the question addresses that the toxic effects of florfenicol on kidney and liver functions in goat, and it’s unclear in literature. Therefore keeping in the view the importance of the subject, this study was designed to evaluate the impact of therapeutic and high doses of florfenicol on liver and kidney functional indicators in goat.

## Materials and Methods

### Ethical approval

The research was approved by Animal Ethics Committee of Sindh Agriculture University, Tandojam as per state laws.

### Experimental Design

Six mature healthy local goats (combine breed and sex) were chosen and averagely weighted as 25 kg. Animals were reared at livestock experimental farm, Sindh Agriculture University, Tandojam, Pakistan. Goats were ear tagged and assigned with A-F letters for identification mark. Goats were fed with green forages; concentrates and water were provided on *ad libitum* basis. The animals were allowed for acclimatization for 21 days before starting of experiment, and during this period animals were dewormetized. Initially, normal or control (C) baseline values of biochemical parameters recognized. The blood samples were drawn aseptically from jugular vein in plane test tubes. The blood samples were transported to the Postgraduate Laboratory of Department of Physiology/Pharmacology, Sindh Agriculture University, Tandojam, Pakistan. The samples were centrifuged at 1500 rpm for 10 min for serum collection. Serum was stored at −20°C for future analysis of biochemical parameters. The absorbance was detected on ultraviolet spectrophotometer according to wavelength mentioned in kits. The serum total protein (TP) quantified through Biuret method (Human Company, Germany). Serum urea measured using urea kit (Human, Germany). Serum creatinine detected via Jaffed reaction method (Human, Germany). Serum alkaline phosphatase (ALP), serum glutamic oxaloacetic transaminase (SGOT or AST), serum glutamic pyruvic transaminase (SGPT or ALT), and gamma-glutamyl transferase (GGT) also calibrated by kit methods (Human, Germany). Serum bilirubin measured by Jendrassik-Grof method (Merck Germany).

### Drug administration and sample collection

Florfenicol (Naflor^©^) obtained from Nawan Laboratory (Karachi, Pakistan) and administered intramuscularly with different dosage regimes. During Phase-I, therapeutic dose (20 mg/kg BW) of florfenicol then in Phase-II first high dose (40 mg/kg BW) and finally in Phase-III second high dose (60 mg/kg BW) induced to goats. Each consecutive dose was administered for 3 day with intervals of 24 h. Blood samples were collected at 0, 24, 48, 72, 96, and 120 h after completion of each dose schedule in each consecutive phase. 0 designated as control (C) exclusive of drug administration. The crossover design with 21 days washout period among the treatments.

### Statistical analysis

All obtained data were represented as means±standard deviation mean and evaluated via one-tailed analysis of variance using student edition statistical program. LSD was used to denote significance difference among the florfenicol induced doses and control baseline at various schedules. p<0.05 was set for significance level.

## Results

### Serum urea

**Figure-1:**
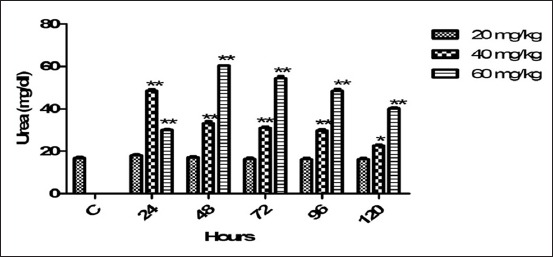
Mean serum urea (mg/dl) level of six goats obtained after intramuscular administration of florfenicol at the dose rate of 20, 40 and 60 mg/kg b.w. Significantly different (*p<0.05, **<0.01) from control values.

Mean values of serum urea with different doses (20, 40, 60 mg/kg BW) of florfenicol at timing points were publicized in Figure-1. Therapeutic dose (20 mg) non-significantly altered the serum urea. At 60 mg dose of florfenicol administration a significant increase (p<0.01) was observed in the serum urea level from 24 to 120 h. The maximum significant increase of serum urea level at this dose was found at 48 h of post drug administration. The concentration of serum urea was statistically increased (p<0.01) through 24-96 h and p<0.05 at 120 h post employed dose of 40 mg.

### Serum creatinine

**Figure-2:**
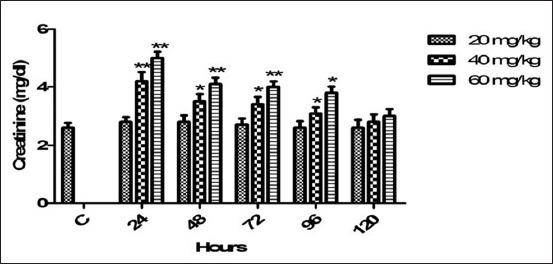
Mean serum creatinine (mg/dl) level of six goats obtained after intramuscular administration of florfenicol at the dose rate of 20, 40 and 60 mg/kg b.w. Significantly different (*p<0.05, **<0.01) from control values.

Mean pre- and post-treated value of creatinine with various doses regimes of florfenicol on programed schedule mentioned in Figure-2. Therapeutic dose non-significantly affected the creatinine level. The concentration of serum creatinine has significantly platuead (p<0.01) from 24 to 72 h and (p<0.05) at 96 h after induction of 60 mg dose. 40 mg dose produced significant (p<0.01) effect via increasing the serum creatinine at 24 h and p<0.05 from 48 to 96 h. The level of creatinine was gradually returned to control baseline value at 120 h.

### Serum TP

**Figure-3:**
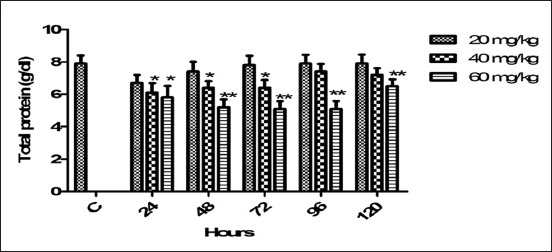
Mean serum total protein (g/dl) level of six goats obtained after intramuscular administration of florfenicol at the dose rate of 20, 40 and 60 mg/kg b.w. Significantly different (*p<0.05, **<0.01) from control values.

As compared to control, the therapeutic dose of florfenicol non-significantly altered the serum TP level. The reduction in serum protein level (p<0.05) was noticed from 24 to 72 h with 40 mg/kg medicated dose of florfenicol (Figure-3). While another high dose (60 mg/kg) produced decreasing effect on the TP at 24 h (p<0.05), and persisted highly significant effect from 48 to 120 h post induction. With 40 mg dose, the serum TP concentration was started to return toward normal values at 96 h.

### Serum ALP

**Figure-4:**
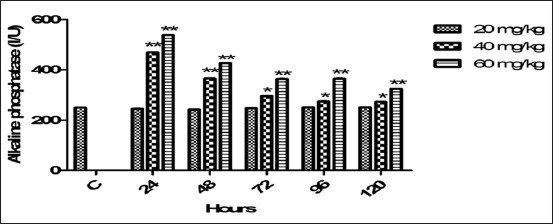
Mean serum alkaline phosphatase (I/U) level of six goats obtained after intramuscular administration of florfenicol at the dose rate of 20, 40 and 60 mg/kg b.w. Significantly different (*p<0.05, **<0.01) from control values.

The average values of ALP treated with therapeutic and high-dosage regimes on various schedules were depicted in Figure-4. 20 mg dose non-significantly altered the serum ALP values. The increased (p<0.01) ALP level was observed since 24-120 h post 60 mg treated dose. While 40 mg dose altered ALP indices (p<0.01) at 24/48 h and (p<0.05) from 72 to 120 h. Both doses produced persistent effect and ALP level was not reversed to control baseline values even up to the 120 h post drug therapy.

### Serum GGT

**Figure-5:**
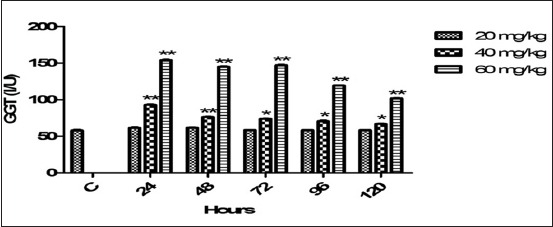
Mean serum gamma-glutamyl transferase (I/U) level of six goats obtained after intramuscular administration of florfenicol at the dose rate of 20, 40 and 60 mg/kg b.w. Significantly different (*p<0.05, **<0.01) from control values.

The mean control and post dosages values of serum GGT on different timing points are mentioned (Figure-5). Non-significantly effect was noticed on GGT with therapeutic dose. The highly significant effects on GGT level was observed in 60 mg dosage regime on all timing points. The GGT level was significantly (p<0.01, <0.05) boosted at all timings followed by dose of 40 mg. The effect of both high doses was persisted, and level of GGT was not backed toward control baseline.

### SGOT

**Figure-6:**
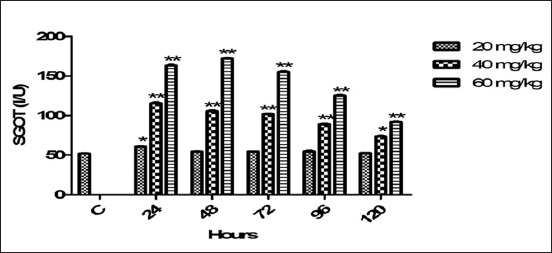
Mean serum glutamic oxaloacetic transaminase (I/U) level of six goats obtained after intramuscular administration of florfenicol at the dose rate of 20, 40 and 60 mg/kg b.w. Significantly different (*p<0.05, **<0.01) from control values.

The average SGOT indices after administration of different doses of florfenicol with various timings publicized in Figure-6. Therapeutic dose significantly (p<0.05) raised SGOT level at 24 h. The both high doses significantly (p<0.01; <0.05) increased the SGOT level all timing points. The high doses persisted effect on SGOT and its level not returned to control indices even up to 120 h post drug medication.

### SGPT

**Figure-7:**
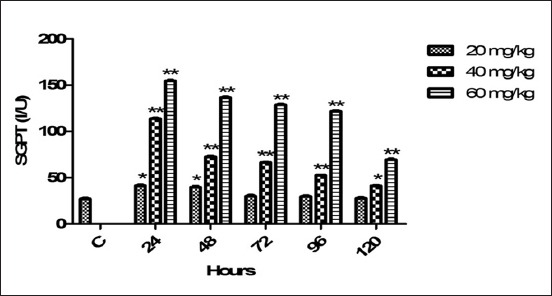
Mean serum glutamate-pyruvate transaminase (I/U) level of six goats obtained after intramuscular administration of florfenicol at the dose rate of 20, 40 and 60 mg/kg b.w. Significantly different (*p<0.05, **<0.01) from control values.

The average SGPT values at various timings medicated with therapeutic and high doses of florfenicol mentioned in Figure-7. In comparison with control, therapeutic dose significantly (p<0.05) increased the SGPT indices at 24 and 48 h. The both high doses raised the SGPT values significantly (p<0.01, <0.05) at all timing points and produced effect persistently.

### Serum bilirubin

**Figure-8:**
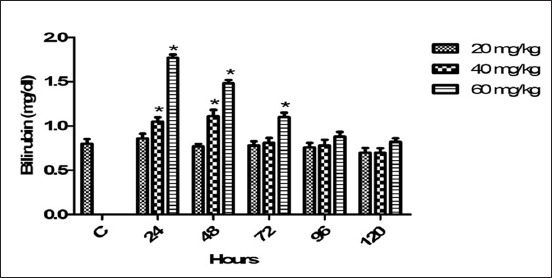
Mean serum bilirubin (mg/dl) level of six goats obtained after intramuscular administration of florfenicol at the dose rate of 20, 40 and 60 mg/kg b.w. Significantly different (*p<0.05) from control values.

The mean serum bilirubin indices after medication of various doses of florfenicol with planned timings had presented in Figure-8. The therapeutic dose non-significantly changed the values of serum bilirubin. Both high dosage regimes significantly (p<0.05) raised the level of bilirubin from 24 to 72 h. The values of bilirubin returned toward control baseline from 96 h.

## Discussion

Therapeutically, florfenicol is extensively practiced in farm animals to cure the infection and also used for prophylaxis as well as feed additive. The main side effects were unusually observed with therapeutic dose (20 mg/kg b.w.) of florfenicol in farm animals. One reported side effect is the elevated bilirubin concentration [10]. By employing high dosage treatment with florfenicol is of great significance to attain valuable plasma and tissues concentration of drug for longer period rather than regular repeated dosing. Although, before using high dosage regimen various side effects of florfenicol on major organs necessarily considered. For that reason, we examined the laboratorial assessment of kidney and liver functional indices. The both high doses of florfenicol elevated the indices of serum urea level which coincide with previously reports [3,11,14]. Whereas, the therapeutic dose produced no effect on urea in this study according to previous report in horse [10]. The urea is produced through deamination of proteins, in the liver and high doses of florfenicol possibly reduced the protein level via triggering the catabolism of protein, and interference with the urea excretion process (glomerular filtration) in kidneys finally retention of urea in the blood [15]. The effect of florfenicol on nitrogen metabolism is probably a reflection of the systemic anti-anabolic action via diminished amino acids incorporation into protein, thus leads an elevation of blood urea [16]. As indicated urea is the soluble and comparatively less toxic end product of protein breakdown formed from ammonia in liver through urea cycle and if protein- nitrogenous and non-protein nitrogenous byproducts not removed from body, they may finally lead to azotemia [11]. The high doses of florfenicol increased serum creatinine levels which were closely matched with previous studies in pig, alpaca, and in fish [11,14,17]. Creatinine is nonprotein nitrogenous product of creatine (phosphocreatine) produced from muscles. Florfenicol might be trigger creatine in muscles leading to elevated level of creatinine in the blood [18]. Another study also confirmed that florfenicol cause degenerative changes in tubules of kidney which ultimately prevent the excretion of creatinine [19]. It is also speculated that florfenicol caused damage to renal tubular cells lead to tubular obstruction, which alter the renal microcirculation finally retention of creatinine level into the blood [20]. Therapeutic dose produced no effect on protein level which coincided with earlier report [10]. High doses of florfenicol decreased the values of serum protein. In microbes, florfenicol inhibit the protein synthesis in the translation stage by binding with specific sites of messenger RNA before exposure to ribosome [21]. The previous studies demonstrated that florfenicol interference with mitochondrial protein synthesis due to similarities between of prokaryotic ribosomal and mitochondrial ribosomal subunits in eukaryotes [22,23]. It is speculated that this similarity may be the base to alter this variable in host, when high doses of florfenicol medicated. Outcomes regarding serum protein level coincide with earlier reports [11,17]. The high doses of florfenicol persistently raised the indices of ALP may be associated with high concentration of drug in the blood. One study confirmed that moderated high doses reversible altered the small magnitude of ALP, which proved that concentration of drug also important [24]. ALP is found in majority of tissues (kidney, bone, and placenta) in the body but more concentrated in liver. It is a membrane-bound enzyme present on biliary epithelial cells and hepatocytes. It is documented that florfenicol induce histological changes in hepatocytes that disturb a cellular permeability process in cell membrane, may allowing the escape of this enzyme into the serum [25]. It is speculated that florfenicol may causes biliary obstruction, and in turn injury to the bile duct epithelium, or cholestasis resulting an increased synthesis of ALP in hepatic cells that lead to its high concentration in serum [26]. During the current study florfenicol elevated the values of GGT. Liver is the primary source of GGT, whereas this enzyme is a membrane-bound protein present on hepatocytes, and biliary epithelia plays a key role in cyclic regeneration of amino acids from extracellular glutathione for synthesis of intracellular glutathione, and cellular detoxification. It is possible florfenicol high concentration may cause cholestasis, then partial obstruction of intrahepatic bile duct and biliary epithelial insult, and ultimately increase in GGT activity [26,27]. Florfenicol might be damage hepatobilliary cells, and elution of damaged membranes triggers the GGT synthesis which in turn cause alteration and increased the half-life of this enzyme [28]. SGPT and SGOT enzymes present in numerous tissues (kidneys, heart, skeletal muscle, brain, and red blood cells) of the body but prominently in liver parenchyma. The high doses of florfenicol produced persistent effect and elevated the SGPT and SGOT levels in the blood due to the presence of high concentration of drug for a longer period. Florfenicol may damage and cause leakage of hepatocytes cell membrane, resulting the releasing of aminotransferases from hepatic cell that leads their increased concentration in blood [29]. Florfenicol may also supposed to induce hepatic hypertrophy leads to induction of hepatic enzymes (functional hypertrophy) and other profound changes that often accompany this phenomenon of increased transaminases [30]. High doses of florfenicol may also be speculated that increased rate of synthesis of SGOT and SGPT altered the half-life of these enzymes in serum [28]. In this study, the results of ALP, GGT, SGPT, and SGOT indices were according to previous documented researches [3,11]. The bilirubin is the breakdown product of normal heme catabolism which is a principal component of red blood cell. Bilirubin plays main physiological role as a cellular antioxidant. In liver bilirubin is conjugated with glucuronic acid via glucuronyl transferase then become soluble bilirubin diglucuronide. It is suggested that elevation of bilirubin was might be the effect of drug with conjugating activity of bilirubin in the liver [10]. Florfenicol may impair hepatic bile flow caused accelerated red blood cells destruction or decreased bilirubin metabolism [31]. The hepato cellular injury and ischemia are other possible reasons for retention of bilirubin into the blood [32]. The increased level of bilirubin matches with previous study [10]. In conclusion, both high doses of florfenicol produced adverse effects on kidney and liver in dose dependent fashion. The outcomes of this study can be guidance for veterinary practitioner in decision making for choosing high dose therapy regarding treatment of bacterial infections. However before usage of high doses, laboratorial analysis of kidney and liver functional indicators must be followed up throughout treatment phase particularly in debilitate farm animals such as goat and sheep.

## Conclusion

It was concluded that the high-dosage regimes of florfenicol produced reversible dose-dependent effects on functional indicators of kidney and liver such as (urea, creatinine, TP, ALP, SGOT, SGPT, GGT, and bilirubin). In the light of these results, it is recommended for the future that high doses of florfenicol evaluated for their effects regarding gross and histopathology of vital organs in goat and other livestock species.

## Authors’ Contributions

JMS was the principle investigator and TAQ was the supervisor. TS and QAS coordinated sampling; MAA and ZAB wrote the manuscript. MS and FAS helped in statistical analysis. All authors review and approve the manuscript for publication.
